# Checkpoint Blockade and Acquired Humoral Immune Dysregulation: Emerging Evidence for Antibody Deficiency During Long-Term PD-1/PD-L1 Inhibition

**DOI:** 10.3390/cancers18152472

**Published:** 2026-08-01

**Authors:** Velizar Shivarov

**Affiliations:** 1 Department of Experimental Research, Medical University Pleven, 5800 Pleven, Bulgaria; vshivarov@abv.bg; 2 Department of Clinical Hematology, St. Sophia General Hospital, 1618 Sofia, Bulgaria

**Keywords:** immune checkpoint inhibitors, PD-1, PD-L1, humoral immunity, class-switch recombination, memory B cells, antibody deficiency, hypogammaglobulinemia, immune-related adverse events, cancer immunotherapy

## Abstract

Immune checkpoint inhibitors have transformed the treatment of multiple malignancies by restoring antitumor T-cell activity. Their clinical identity is therefore that of immune-enhancing therapies. However, the biology of the PD-1/PD-L1 axis is more complex than simple immune inhibition. Human inborn errors of PD-1 or PD-L1 signaling indicate that this pathway contributes to immune homeostasis, tolerance, protection against selected infections, and the development of memory B cells and antibody responses. These observations raise an important translational question: Can prolonged pharmacologic blockade of PD-1 or PD-L1 can, in selected clinical contexts, induce or reveal acquired humoral immune dysfunction? This review synthesizes evidence linking PD-1/PD-L1 disruption to altered class-switched memory B-cell biology, antibody responses, vaccine immunogenicity, infection susceptibility, and secondary antibody deficiency. It also incorporates emerging evidence that checkpoint blockade may expand age-associated B cells, a population associated with impaired neutralizing antibody responses after vaccination, and counterbalances evidence that vaccination during ICI therapy may enhance antitumor immunity and survival. Current clinical evidence does not establish the incidence, prevalence, reversibility, dose dependence, or causality of an ICI-induced antibody-deficiency syndrome. Instead, the available data support a hypothesis-generating model of heterogeneous humoral remodeling, ranging from preserved or enhanced vaccine-associated immune activation to qualitative antibody failure and secondary antibody deficiency in susceptible patients. Future studies should incorporate baseline and longitudinal measurements of immunoglobulins, vaccine-specific and neutralizing antibodies, class-switched memory B cells, age-associated B cells, plasmablasts, infection burden, and exposure to immunosuppressive treatment.

## Simple Summary

Immune checkpoint inhibitors are cancer treatments that strengthen antitumor immune responses. However, the same immune pathways may also be important for maintaining normal antibody responses. Recent studies of people with inherited PD-1 or PD-L1 deficiency suggest that this pathway is involved in memory B-cell development and antibody production. This review discusses whether long-term PD-1/PD-L1 blockade could, in selected patients, contribute to abnormal humoral immunity, impaired vaccine quality, recurrent infections, or secondary antibody deficiency. Current evidence does not prove that checkpoint inhibitors commonly cause a CVID-like syndrome. Instead, the available data support a hypothesis-generating model of humoral immune remodeling, ranging from preserved or enhanced vaccine-associated immune activation to qualitative antibody dysfunction in susceptible patients. We propose a practical framework for future studies and for risk-adapted clinical evaluation rather than universal immune screening.

## 1. Introduction

Immune checkpoint inhibitors (ICIs) have reshaped modern oncology by producing durable responses in melanoma, non-small-cell lung cancer, renal-cell carcinoma, urothelial carcinoma, mismatch-repair-deficient tumors, and several other malignancies [1,2]. Antibodies targeting CTLA-4, PD-1, or PD-L1 are often described as immune-enhancing therapies because they attenuate inhibitory signaling pathways that restrain antitumor T-cell function [3,4]. This framing is clinically useful, but it is biologically incomplete. PD-1 and PD-L1 are not only tumor-immune escape molecules. They are also central regulators of immune homeostasis, tissue tolerance, T-cell differentiation, and adaptive immune architecture [4,5].

The long-term immunologic consequences of checkpoint blockade are becoming increasingly relevant. ICIs are now used earlier in cancer care, including adjuvant, neoadjuvant, consolidation, and maintenance settings. As survival improves, more patients are exposed for months or years, and more survivors live with persistent immune perturbation after treatment cessation [6,7]. Chronic and late immune-related adverse events (irAEs) are increasingly recognized, but most clinical attention has focused on inflammatory organ toxicities such as colitis, pneumonitis, hepatitis, endocrinopathies, nephritis, arthritis, myocarditis, neurologic syndromes, and hematologic autoimmunity [6,8]. By contrast, the possibility that checkpoint blockade may induce, unmask, or amplify clinical manifestations of immune deficiency has received far less attention.

This question is especially relevant for humoral immunity. Durable protection against encapsulated bacteria, respiratory viruses, herpesviruses, and vaccine-preventable pathogens depends on coordinated germinal-center reactions, class-switch recombination (CSR), affinity maturation, plasma-cell generation, and memory B-cell maintenance [9,10,11,12]. The PD-1 pathway is active in germinal-center biology: T follicular helper (Tfh) cells express high levels of PD-1, and PD-L1 is expressed by antigen-presenting cells, B cells, and non-hematopoietic tissues [13,14]. Although PD-1 signaling is usually interpreted as inhibitory, inherited PD-1/PD-L1 deficiency and recent mechanistic work indicate that the same pathway may also support effective antibody responses through B-cell-intrinsic and B-cell-extrinsic mechanisms [15,16].

**Figure 1:**
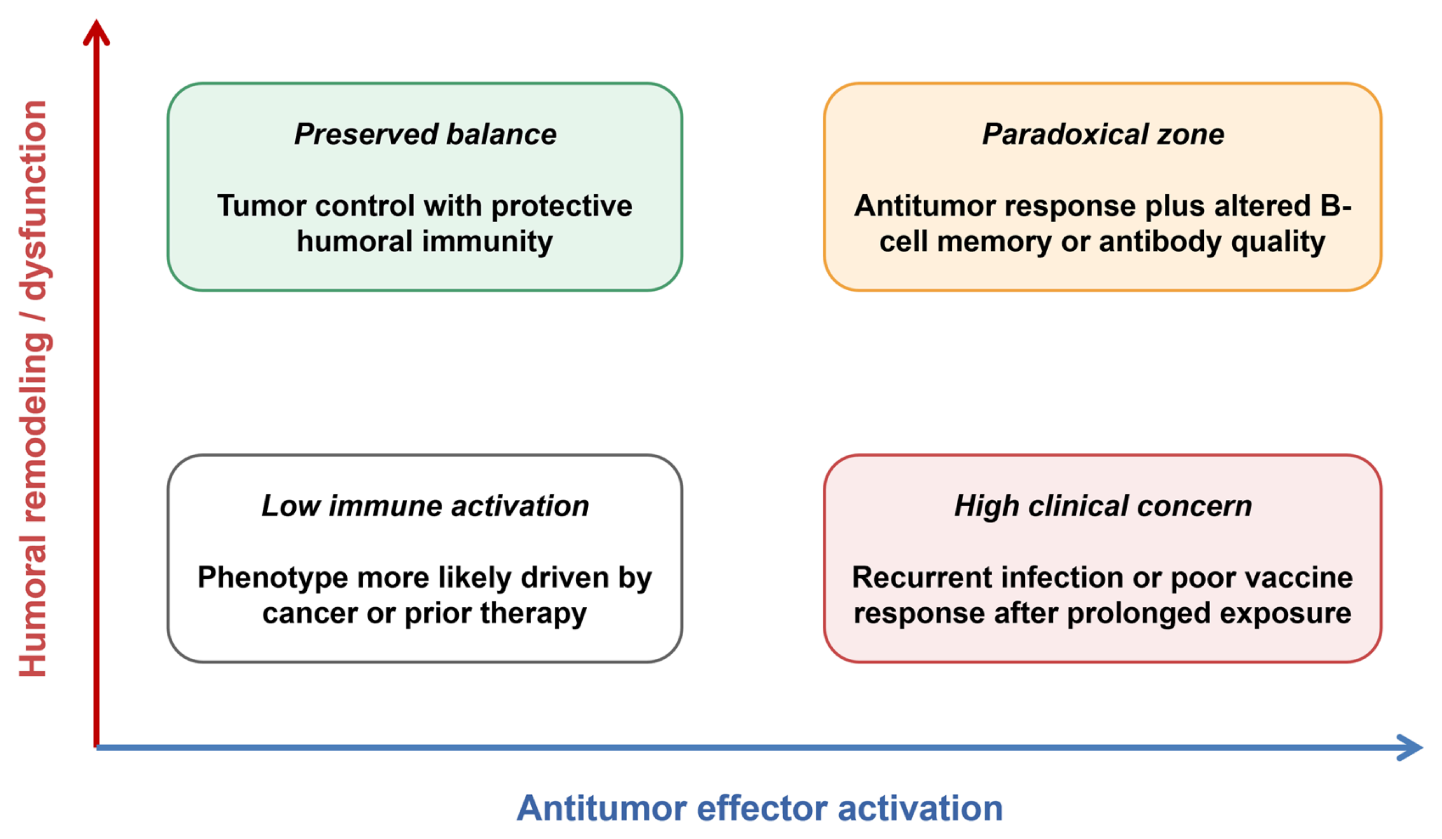
Conceptual two-dimensional model of humoral immune remodeling during immune checkpoint inhibition. This schematic summarizes the proposed relationship between antitumor effector activation and humoral immune remodeling or dysfunction during long-term immune checkpoint inhibitor therapy. The horizontal axis represents increasing antitumor effector activation, primarily mediated through restoration of exhausted antitumor T-cell responses. The vertical axis represents increasing humoral immune remodeling or dysfunction, including altered B-cell memory, impaired antibody quality, poor vaccine response, or recurrent infection. The upper-left quadrant represents preserved balance, in which tumor control occurs together with protective humoral immunity. The upper-right “paradoxical zone” represents the central hypothesis of this review: antitumor benefit may coexist with altered B-cell memory or antibody quality. The lower-left quadrant represents low immune activation, where immune phenotypes are more likely to be driven by the underlying cancer, prior therapy, or other non-ICI-related factors. The lower-right quadrant represents a high-clinical-concern phenotype, in which recurrent infections or poor vaccine responses arise after prolonged exposure and should prompt risk-adapted humoral immune evaluation. ICI, immune checkpoint inhibitor.

The central thesis of this review is that long-term PD-1/PD-L1 blockade may create a paradoxical and heterogeneous immune state: enhanced antitumor effector immunity on one axis and altered humoral architecture on another (Figure 1). This should not be interpreted as global immunosuppression. The emerging signal is better described as humoral remodeling, in which some patients retain effective vaccine responses or derive antitumor benefit from vaccine-associated immune activation, whereas others may develop impaired neutralization, reduced antigen-specific memory, expansion of dysfunctional B-cell compartments, recurrent infections, or secondary antibody deficiency [17,18].

For the purposes of this review, “humoral immune remodeling” refers to treatment-context-associated changes in B-cell composition, antibody function, or immune memory that do not necessarily fulfill diagnostic criteria for a primary antibody deficiency. This concept includes quantitative abnormalities, such as declining IgG, IgA, or IgM levels; cellular abnormalities, such as reduced class-switched memory B cells, altered plasmablast responses, expansion of CD21low or age-associated B cells (ABCs); and functional abnormalities, such as impaired vaccine-specific neutralization, reduced antibody durability, or recurrent infections compatible with defective humoral protection. The term is intentionally broader than “antibody deficiency” because current evidence does not establish that PD-1/PD-L1 blockade commonly causes a reproducible CVID-like syndrome. Instead, it provides a framework for studying heterogeneous B-cell and antibody changes in selected patients exposed to long-term checkpoint inhibition.

This is a narrative review. The literature was identified through targeted searches of PubMed and Google Scholar using combinations of “PD-1 deficiency,” “PD-L1 deficiency,” “immune checkpoint inhibitor,” “humoral immunity,” “memory B cells,” “class-switch recombination,” “hypogammaglobulinemia,” “secondary antibody deficiency,” “infection,” “vaccination,” “age-associated B cells,” and “immune-related adverse events.” Priority was given to human genetic studies, mechanistic immunology studies, clinical vaccine studies, infection analyses, pharmacovigilance studies, guidelines, and recent reviews. This approach was intended to synthesize a developing concept rather than to provide a systematic review or meta-analysis.

## 2. Lessons from Inherited PD-1 and PD-L1 Deficiency

The strongest biological evidence comes from human inborn errors of PD-1/PD-L1 signaling. These rare conditions provide a natural experiment, but they must not be treated as direct clinical equivalents of therapeutic checkpoint blockade. Inherited PD-1 deficiency was described in a child with severe immune dysregulation, autoimmunity, and tuberculosis [19]. The patient developed early-onset autoimmune manifestations and eventually fatal pulmonary autoimmunity. The leukocytes did not express PD-1 and did not respond to PD-1-mediated suppression [19]. This phenotype showed that lifelong absence of PD-1 does not produce uniformly beneficial immune activation. Instead, PD-1 deficiency causes immune dysregulation, with failure to balance pathogen control, tissue tolerance, and inflammatory restraint.

Inherited PD-L1 deficiency has since been reported in siblings with neonatal-onset type 1 diabetes caused by biallelic CD274 loss of function [15]. Compared with PD-1 deficiency, PD-L1 deficiency appeared clinically and immunologically less severe. This difference may reflect partial compensation through PD-L2 or broader receptor-ligand effects when PD-1 itself is absent [15]. The distinction is highly relevant for oncology because anti-PD-1 and anti-PD-L1 antibodies may not be biologically identical in their effects on B-cell activation, Tfh help, and antibody durability.

The finding most directly relevant to this review is that inherited PD-1 or PD-L1 deficiency is associated with impaired memory B-cell development and attenuated antibody responses [16]. Affected individuals had fewer memory B cells, abnormalities in class-switched memory compartments, and impaired antibody responses to environmental microbial antigens [16]. Parallel mouse experiments showed similar defects, and B-cell-specific deletion of Pdcd1 impaired physiologic accumulation of memory B cells [16]. Mechanistically, PD-1- or PD-L1-deficient human B cells showed reduced expression of c-Myc and c-Myc target genes, while experimental disruption of PD-1/PD-L1 signaling impaired antibody production by activated human B cells [16].

These findings support a reinterpretation of the PD-1 axis. PD-1/PD-L1 signaling is not merely a brake on lymphocyte activation. In B-cell biology, it may function as a context-dependent support system for activation, differentiation, and memory formation [16,20]. However, the inherited-deficiency phenotype should be used as a mechanistic anchor rather than as proof that adult cancer patients receiving ICIs develop the same condition (Table 1).

Human PD-1/PD-L1 deficiency and related B-cell remodeling evidence is summarized in Table 2.

## 3. PD-1/PD-L1 Signaling, Class-Switch Biology, and Memory B-Cell Formation

Class-switch recombination (CSR) is the DNA recombination process through which activated B cells replace IgM/IgD constant regions with IgG, IgA, or IgE isotypes while preserving antigen specificity [12,22,23,24]. CSR requires B-cell receptor stimulation, CD40-CD40L interaction, cytokine signaling, activation-induced cytidine deaminase (AID), double-stranded DNA breaks, proliferation, metabolic competence, and appropriate T-cell support [12,22,23,24,25,26,27]. Dysregulation of these processes can manifest as hyper-IgM syndromes, CVID-like phenotypes, impaired vaccine responses, reduced class-switched memory B cells, or complex immune dysregulation [22,25,26,27].

Current evidence does not show that PD-1/PD-L1 deficiency is a classical monogenic CSR defect. Available human data do not establish defective switch-junction formation, AID failure, or impaired germline transcription as the primary mechanism. The term “CSR deficiency” should therefore be used cautiously. A more precise formulation is that PD-1/PD-L1 disruption is associated with a class-switched memory B-cell and antibody-response defect. This may involve impaired CSR, reduced survival of switched cells, defective differentiation, altered germinal-center selection, or attrition after differentiation [13,16,28,29,30].

### 3.1. PD-1/PD-L1 as Both Brake and Support

The apparent contradiction between PD-1 as an inhibitory receptor and PD-1/PD-L1 signaling as a support pathway for humoral immunity can be resolved by considering cell type, timing, and anatomical context. In effector T cells, PD-1 signaling restrains activation and tissue injury. In the germinal-center environment, however, PD-1/PD-L1 interactions may help regulate the quantity, localization, and quality of Tfh-B-cell collaboration. Excessive inhibition may limit antibody generation, but complete or prolonged disruption may also impair the organized selection and differentiation required for durable memory. Thus, checkpoint blockade may not simply increase humoral help; it may alter the architecture of help, selection, and memory formation.

Tfh cells provide essential signals to germinal-center B cells, including CD40 ligand, interleukin-21, interleukin-4, ICOS-dependent interactions, and antigen-specific selection cues [13,28,30]. PD-1 is highly expressed on Tfh cells, particularly in germinal centers. In some models, PD-1 restrains excessive Tfh help and autoantibody production [13,14,28]. Yet excessive or absent PD-1 signaling may both be harmful, depending on context. Physiologic PD-1/PD-L1 signaling may help organize the spatial and quantitative delivery of Tfh help, whereas complete or sustained disruption may destabilize germinal-center output.

### 3.2. B-Cell-Intrinsic Signaling and c-Myc

**Figure 2:**
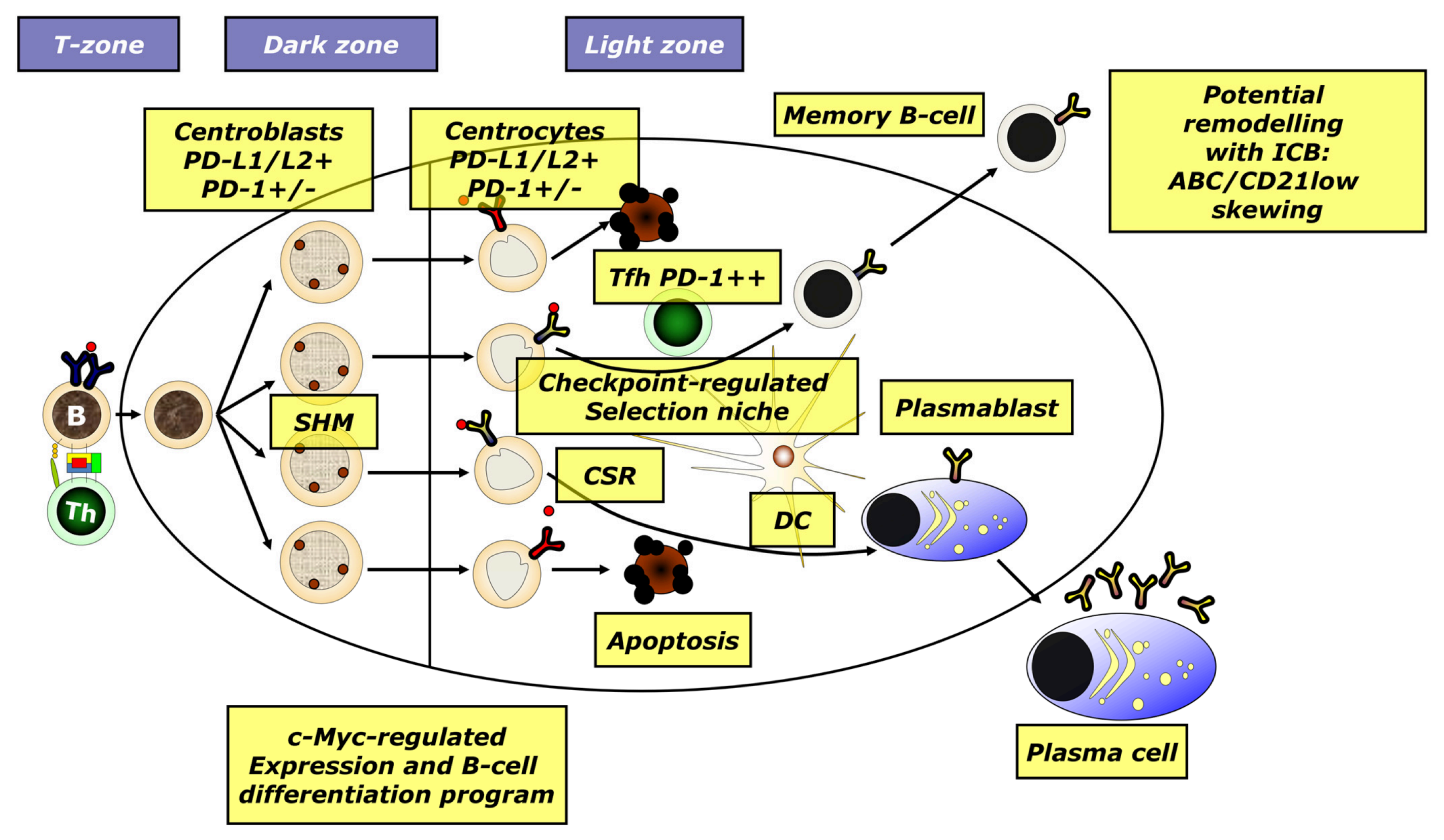
Proposed localization of PD-1/PD-L1 pathway activity within the germinal-center reaction and its potential relationship to humoral remodeling. The figure illustrates the germinal-center pathway from initial T-zone B-cell and helper T-cell interaction through the dark zone, light zone, selection, class-switch recombination, somatic hypermutation, memory B-cell generation, plasmablast formation, and plasma-cell differentiation. T follicular helper cells in the light zone are characterized by high PD-1 expression and provide essential help for antigen-specific B-cell selection. Germinal-center B-cell compartments, including centroblasts and centrocytes, are depicted as engaging the PD-L1/PD-L2 ligand interface, with PD-1 expression shown as variable or subset-dependent rather than uniformly expressed. The checkpoint-regulated selection niche highlights the interaction between PD-1-high Tfh cells, PD-L1/PD-L2-expressing B cells or antigen-presenting cells, and dendritic-cell-supported antigen presentation. The c-Myc-regulated activation and differentiation program indicates a proposed mechanistic link between PD-1/PD-L1 signaling, B-cell metabolic activation, proliferation, antibody production, and memory formation. Potential remodeling with immune checkpoint blockade is shown as ABC/CD21low skewing near the memory B-cell output, reflecting an atypical memory-like B-cell phenotype associated with reduced antigen-specific memory and impaired neutralizing antibody responses in some settings. ABC, age-associated B cell; CD21low, B cells with low CD21 expression, often overlapping with atypical memory-like B cells; CSR, class-switch recombination; DC, dendritic cell; ICB, immune checkpoint blockade; PD-1, programmed cell death protein 1; PD-L1, programmed death-ligand 1; PD-L2, programmed death-ligand 2; SHM, somatic hypermutation; Tfh, T follicular helper cell.

B-cell-intrinsic PD-1/PD-L1 signaling is an additional regulatory layer (Figure 2). Human naive B cells can express PD-1 and/or PD-L1 after activation, and PD-1/PD-L1 disruption can impair c-Myc expression and antibody production in vitro [16]. c-Myc is central to lymphocyte growth, proliferation, metabolic reprogramming, and differentiation. Defective c-Myc induction provides a plausible mechanistic bridge between checkpoint signaling and impaired humoral output. The relative contribution of direct B-cell signaling versus altered Tfh help remains unresolved, but this uncertainty defines testable experiments for ICI-treated cohorts.

### 3.3. Age-Associated B Cells and Qualitative Humoral Failure During Checkpoint Blockade

Age-associated B cells (ABCs), also described as CD11c+ T-bet+ or atypical memory-like B cells, are an important addition to the mechanistic model. ABCs accumulate with aging, chronic immune stimulation, autoimmunity, inborn errors of immunity, and cancer immunotherapy. Yam-Puc et al. showed that patients receiving immune checkpoint blockade can exhibit premature ABC expansion and that higher baseline ABC frequency correlated with reduced antigen-specific memory B cells and reduced neutralizing capacity after COVID-19 vaccination [21]. This shifts the proposed phenotype from simple quantitative antibody deficiency to qualitative humoral failure: patients may produce binding antibodies yet generate weaker neutralization, poorer durability, or less effective antigen-specific memory.

ABCs should therefore be treated as candidate biomarkers of dysfunctional humoral remodeling in future ICI cohorts. The diagnostic and research framework should avoid exclusive reliance on total IgG or seroconversion. It should include ABCs/CD21low B cells, antigen-specific memory B cells, neutralization assays, durability of vaccine-specific antibodies, and, where feasible, B-cell receptor repertoire or somatic hypermutation analyses [21].

These mechanisms establish why PD-1/PD-L1 signaling could plausibly affect humoral immunity. However, they do not prove that therapeutic checkpoint blockade reproduces inherited deficiency. The next section therefore separates what can be inferred from genetic and mechanistic models from what remains uncertain in adult cancer patients exposed to intermittent pharmacologic blockade.

## 4. What Can and Cannot Be Inferred from Pharmacologic PD-1/PD-L1 Blockade?

A major interpretive challenge is whether lifelong inherited deficiency can be extrapolated to adult pharmacologic blockade. The extrapolation is only partially valid. Inherited deficiency affects immune development from birth, whereas ICIs are usually administered to adults with an already formed immune system. Monogenic PD-1 or PD-L1 deficiency is complete or near-complete, while therapeutic blockade is intermittent, dose-dependent, tissue-dependent, and influenced by drug pharmacokinetics. Cancer patients also differ from genetically deficient children because they may have malignancy-related immune dysfunction, prior chemotherapy, radiation, targeted therapy, surgery, malnutrition, infection exposure, and age-related immune remodeling [31,32].

The comparison remains analytically useful because both settings disrupt the same signaling axis. Long-term ICI therapy can produce persistent immune effects, and chronic irAEs may continue after drug discontinuation [7,33]. Pharmacologic blockade may be most relevant when new humoral responses must be generated during therapy, such as vaccination, viral infection, bacterial infection, tumor-antigen recognition, or tissue autoimmunity. An adult with intact pre-existing memory may seroconvert after vaccination, yet the quality, breadth, affinity, or durability of the response could still change. This distinction helps explain why many vaccine studies are reassuring but do not exclude subtler long-term defects.

Inherited PD-1/PD-L1 deficiency should therefore be interpreted as a biological warning signal and mechanistic model, not as definitive evidence that ICIs typically induce antibody deficiency. The current clinical literature does not establish incidence, prevalence, reversibility, dose dependence, or causality. It also does not adequately resolve whether anti-PD-1 and anti-PD-L1 therapies have different humoral consequences. The hypothesis requires prospective studies that distinguish direct checkpoint effects from indirect effects of irAE treatment, because high-dose corticosteroids and second-line immunosuppressants can themselves cause clinically meaningful secondary immune deficiency.

## 5. Vaccine Responses During Immune Checkpoint Blockade

Vaccination studies provide the most direct clinical window into humoral immunity during ICI therapy. Influenza vaccine studies generally show that patients receiving PD-1/PD-L1 blockade can mount serologic responses, although magnitude, cellular correlates, and safety signals vary [18,31]. Herati et al. showed that PD-1-directed immunotherapy altered Tfh and humoral immune responses to seasonal influenza vaccination, with more robust circulating Tfh responses in a subset of adults receiving anti-PD-1 therapy [17]. The study also found transcriptional evidence of increased proliferation in responding Tfh and B-cell compartments, supporting the idea that PD-1 blockade modulates the Tfh-B-cell axis rather than simply preserving its basal state [17].

Earlier influenza vaccine investigations suggested serological protection in a significant proportion of ICI-treated patients, although concerns about increased irAEs were raised [31]. Subsequent systematic analysis has generally supported the safety and immunogenicity of influenza vaccination during ICI therapy, but heterogeneity remains substantial [18]. These findings are reassuring clinically, yet most studies were designed to determine whether vaccination is safe and whether short-term titers rise, not whether germinal-center output is quantitatively and qualitatively normal.

COVID-19 vaccine studies similarly argue against a simple global failure of antibody production during ICI therapy. Systematic reviews and meta-analyses indicate that many patients receiving ICIs seroconvert after SARS-CoV-2 vaccination, often with responses superior to those observed during cytotoxic chemotherapy and broadly comparable to some cancer-control cohorts [34,35]. However, these studies vary in timing, cancer type, prior therapy, vaccine platform, variant exposure, antibody assay, and immune endpoints [34,35]. Many did not include pre-treatment immunoglobulins, class-switched memory B cells, neutralization breadth, mucosal IgA, or long-term durability.

The key distinction is that seroconversion is not equivalent to complete humoral competence. Binding antibodies, neutralizing antibodies, class-switched memory B cells, durability, and clinical protection are related but not interchangeable endpoints. Current vaccine research therefore supports a nuanced interpretation: ICIs do not generally abolish antibody responses, but they may reshape the cellular and regulatory context in which humoral responses develop [17,21,31,34].

### Vaccination-Associated Immune Potentiation and Survival Benefit

The dysregulation hypothesis must also be reconciled with evidence that vaccination during ICI therapy may improve antitumor immunity. Grippin et al. reported that SARS-CoV-2 mRNA vaccination within 100 days of ICI initiation was associated with improved median and three-year overall survival in large retrospective cohorts and provided mechanistic evidence that mRNA vaccination can induce type I interferon, myeloid–lymphoid activation, tumor PD-L1 induction, and enhanced ICI sensitivity [36]. These findings argue against a simplistic model in which ICI-treated patients are globally humoral-immunodeficient. Instead, they support a dual model: acute vaccine-associated innate and T-cell immune potentiation may enhance tumor control, while selected patients may still display ABC-associated qualitative antibody failure, reduced neutralization, or impaired memory durability [21,36].

Importantly, several vaccine and ABC studies are observational and cannot completely exclude confounding by age, tumor type, disease stage, prior therapy, infection history, vaccine platform, corticosteroid exposure, or healthy-user effects. They should therefore be interpreted as evidence of altered immune dynamics, not as direct proof that PD-1/PD-L1 blockade causes clinically meaningful antibody deficiency.

## 6. Infection Risk After Checkpoint Inhibition

The infection literature is multifaceted. Unlike chemotherapy, anti-CD20 therapy, BTK inhibition, CAR-T therapy, or bispecific T-cell engagers, ICIs do not cause predictable neutropenia or direct B-cell depletion [37]. Reviews suggest that ICIs themselves do not broadly increase infection risk in a conventional immunosuppressive manner [37]. However, infections are common in ICI-treated patients because cancer, comorbidities, barrier injury, lung disease, prior therapies, hospital exposure, antibiotics, and irAE-directed immunosuppression all contribute [37].

Several infection patterns are relevant. Tuberculosis and atypical mycobacterial infections have been reported during PD-1/PD-L1 blockade, consistent with the role of PD-1 in mycobacterial immunity and with the PD-1-deficient human phenotype [19]. Viral infections, including herpesviruses, hepatitis reactivation, and severe respiratory viral infections, have been described in ICI-treated populations, although causality is difficult to establish [37]. Pharmacovigilance studies report signals for pneumonia, sepsis, tuberculosis, atypical mycobacterial infections, and other infectious adverse events during ICI therapy [38]. These analyses are useful for signal detection but cannot estimate true incidence or identify mechanism.

Infections are therefore best interpreted as clinical readouts rather than direct proof of humoral remodeling. A remodeling phenotype is most plausible when infections are recurrent, microbiologically documented, predominantly sinopulmonary, caused by encapsulated bacteria or vaccine-preventable pathogens, temporally associated with new abnormalities in immunoglobulins or vaccine-specific antibodies, and accompanied by reduced class-switched memory B cells or impaired neutralization. In contrast, isolated pneumonia during pneumonitis, sepsis during hospitalization, viral reactivation after high-dose corticosteroids, or opportunistic infection after additional immunosuppression is more consistent with cancer-related or treatment-related secondary immunodeficiency. This distinction should guide clinical interpretation and the design of future studies.

**Figure 3:**
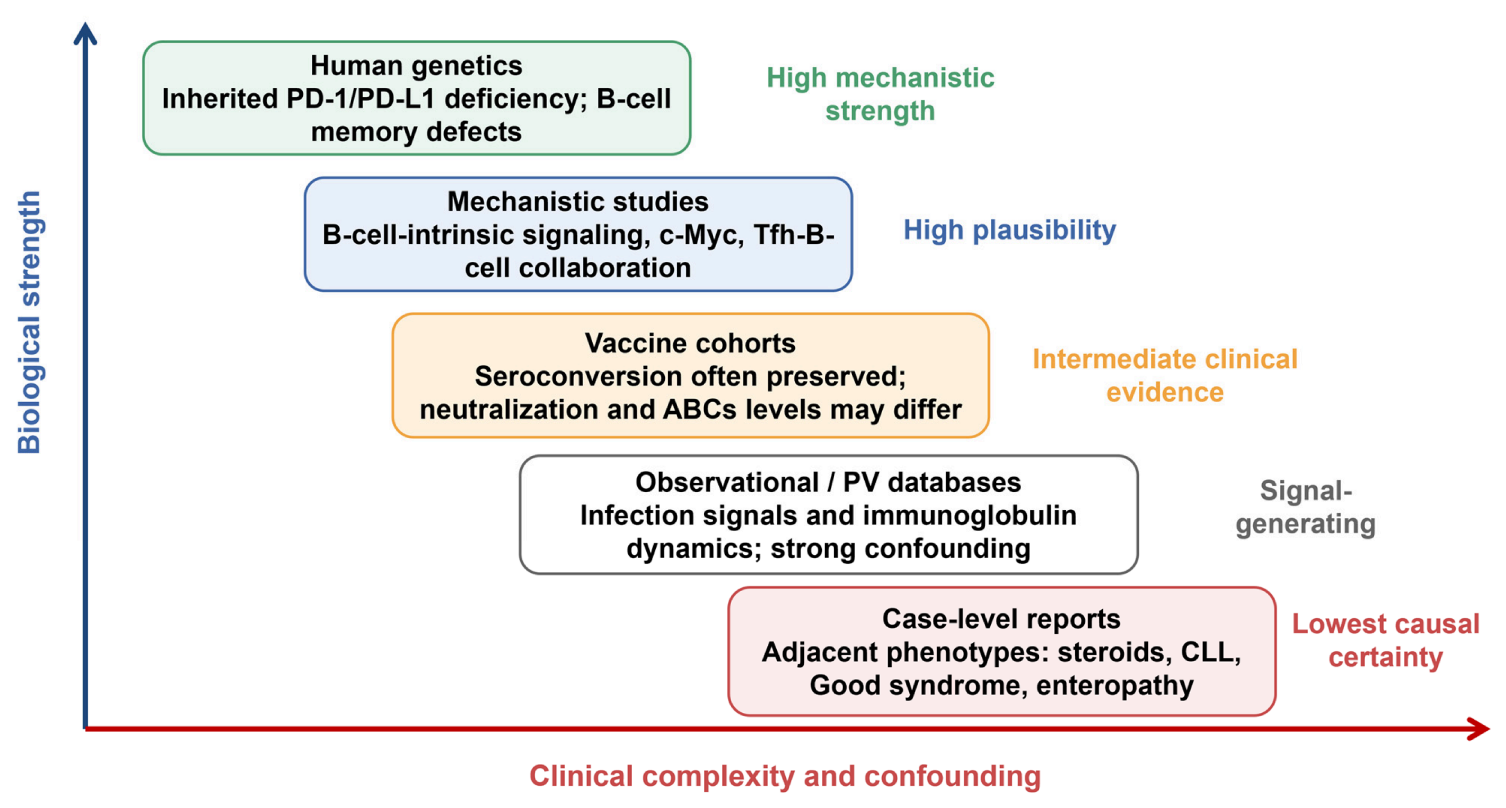
Evidence map linking biological plausibility to clinical uncertainty in ICI-associated humoral immune remodeling. This schematic ranks major evidence domains according to biological strength and clinical complexity. Human genetic evidence from inherited PD-1 or PD-L1 deficiency provides high mechanistic strength because these disorders demonstrate immune dysregulation and B-cell memory defects when the pathway is disrupted. Mechanistic human and murine studies provide high biological plausibility by implicating B-cell-intrinsic signaling, c-Myc regulation, and Tfh–B-cell collaboration in memory B-cell and antibody-response development. Vaccine cohorts provide intermediate clinical evidence: seroconversion is often preserved, but neutralizing capacity, antibody durability, antigen-specific memory B cells, and ABC frequencies may differ across patient subsets. Observational studies and pharmacovigilance databases provide signal-generating evidence for infections or immunoglobulin dynamics but are strongly confounded by cancer type, disease stage, prior therapy, age, corticosteroids, and treatment of immune-related adverse events. Case-level reports provide the lowest causal certainty because most describe adjacent or confounded phenotypes, including corticosteroid-associated immune deficiency, chronic lymphocytic leukemia, Good’s syndrome, protein loss, enteropathy, or prior immunosuppressive therapy. ABC, age-associated B cell; CLL, chronic lymphocytic leukemia; ICI, immune checkpoint inhibitor; PD-1, programmed cell death protein 1; PD-L1, programmed death-ligand 1; PV, pharmacovigilance; Tfh, T follicular helper cell.

A humoral-immunity-focused infection analysis should therefore ask specific questions. Are infections recurrent or opportunistic? Are they predominantly sinopulmonary? Are encapsulated bacteria overrepresented? Are IgG, IgA, IgM, IgG subclasses, and vaccine-specific antibodies reduced? Are class-switched memory B cells low? Did the patient receive steroids, infliximab, mycophenolate, rituximab, chemotherapy, or anti-B-cell therapies? Without these data, the infection literature remains suggestive but mechanistically unresolved (Figure 3).

## 7. Secondary Antibody Deficiency After ICIs: Case-Level Evidence and Confounders

Direct case-level evidence for checkpoint blockade-induced secondary antibody deficiency remains limited. The most relevant reports illustrate confounded or adjacent phenotypes, including secondary antibody deficiency associated with endogenous hypercortisolism, hypogammaglobulinemia in patients with CLL or thymoma/Good’s syndrome who subsequently received ICIs, immune-mediated enteropathy after pembrolizumab exposure with secondary immunologic consequences, and measurement of immunoglobulin dynamics during ICI therapy [39,40,41,42,43]. Together, these observations support the plausibility and clinical importance of humoral immune monitoring in selected patients, but they do not define a reproducible syndrome of ICI-induced acquired antibody deficiency.

Interpretation is difficult because patients may have received chemotherapy, radiation, targeted therapy, corticosteroids, anti-CD20 antibodies, or other immunosuppressants before or after ICI exposure. Underlying malignancy can also impair humoral immunity, particularly in hematologic cancers, thymic epithelial tumors, and heavily pretreated solid tumor populations [44,45]. A risk-adapted diagnostic protocol is summarized in Table 3. This work distinguishes four clinical categories (Table 4): direct checkpoint-blockade-associated humoral dysregulation; irAE-treatment-associated secondary antibody deficiency; cancer- or prior-treatment-associated immune deficiency; and unmasked primary immune dysregulation [46].

Although this classification can be mapped to established immunological nomenclature, it requires caution. ESID criteria for CVID and unclassified antibody deficiency emphasize recurrent or clinically relevant immune manifestations, low IgG with low IgA and/or IgM, impaired vaccine responses and/or reduced isotype-switched memory B cells, absence of profound T-cell deficiency, and exclusion of secondary causes [47]. ICI-associated humoral dysfunction should not be labeled CVID unless it fulfills primary-immunodeficiency criteria and predates or exceeds treatment effects. For most oncology patients, the more appropriate framework is secondary hypogammaglobulinemia or secondary antibody deficiency, in which medication exposure, malignancy, protein loss, or other acquired processes reduce immunoglobulin quantity or antibody function [48].

## 8. Chronic and Late Immune Toxicity as a Survivorship Problem

The long-term dimension is critical because anti-PD-1/PD-L1 therapies are now given in settings where patients may be cured or live for many years. Chronic irAEs are frequent but heterogeneous and should not be described as universally permanent. In the Patrinely et al. adjuvant melanoma cohort, chronic irAEs occurred in 167 of 387 patients (43.2%), were mostly grade 1–2, and persisted at last follow-up in 143 of 167 affected patients (85.6%) [49]. Extended follow-up by Goodman et al. showed partial resolution over time, with persistent chronic irAEs in 63.3% of affected patients, corresponding to 29.2% of all anti-PD-1-treated patients [50]. These data support long-term immune perturbation but not absolute permanence for all toxicity classes.

Humoral immune monitoring is not part of routine ICI survivorship. Current oncology standards emphasize structured management of immune-related toxicities, immunization, survivorship care coordination, and risk-adapted infection prevention, but they do not establish universal serial immunoglobulin or B-cell-subset monitoring for all ICI-treated survivors [51,52]. Therefore, the monitoring framework proposed here should be presented as a targeted clinical evaluation and research strategy for patients with recurrent infections, poor vaccine responses, prolonged corticosteroid or second-line immunosuppressive exposure, hematologic malignancy, prior B-cell-directed therapy, or other features suggesting secondary antibody deficiency.

In practical terms, quantitative immunoglobulins and vaccine-specific antibodies are most defensible when there are recurrent sinopulmonary infections, unusual or severe infections, poor vaccine durability, prolonged systemic immunosuppression, hypoproteinemia or protein loss, unexplained lymphopenia, or a prior history suggestive of immune dysregulation [48,51,52,53]. This risk-adapted approach aligns with current cancer survivorship and vaccination guidance while acknowledging that available evidence does not justify universal screening.

## 9. Evidence Strength, Confounding, and Limitations

The evidence base supporting ICI-associated humoral remodeling is uneven. Human genetic data and mechanistic studies provide strong biological plausibility, but clinical causality remains unproven. Vaccine studies show that humoral responses are often preserved and may be immunologically enhanced in some contexts, while ABC/neutralization studies suggest that antibody quality may differ in selected patients. Infection studies and pharmacovigilance analyses are useful for signal generation but are strongly confounded by malignancy, prior therapies, organ toxicity, hospitalization, antibiotic exposure, and irAE-directed immunosuppression. Case reports are important for hypothesis generation but cannot define incidence or causality.

Several limitations should therefore be emphasized. Current evidence does not establish the incidence or prevalence of humoral remodeling after ICIs, its dose or duration dependence, its reversibility after discontinuation, or whether anti-PD-1 and anti-PD-L1 antibodies differ clinically. Most studies lack baseline immunoglobulins, pre-treatment vaccine-specific antibody titers, standardized B-cell phenotyping, neutralization assays, mucosal IgA assessment, and adjudicated infection outcomes. Age-related immune remodeling, cancer type and stage, chemotherapy, anti-CD20 therapy, hematologic malignancy, corticosteroids, mycophenolate, infliximab, rituximab, protein loss, and pre-existing immune dysregulation must be treated as major alternative explanations.

The proposed framework should therefore be read as a testable model and clinical research agenda, not as evidence that PD-1/PD-L1 blockade commonly causes clinically meaningful antibody deficiency. This distinction is essential to avoid overdiagnosis and unnecessary screening while still recognizing a biologically plausible survivorship issue that merits prospective evaluation. The hierarchy of evidence is summarized in Table 5.

## 10. Research Agenda

Prospective studies should be designed specifically to address humoral immunity. A minimum dataset should include pre-treatment and serial IgG, IgA, IgM, IgG subclasses, lymphocyte subsets, naive B cells, unswitched memory B cells, class-switched memory B cells, plasmablasts, CD21low B cells, ABCs/CD11c + T-bet + B cells, Tfh-like circulating CD4 subsets, and vaccine-specific antibody titers. Because qualitative failure may occur despite detectable binding antibody, future studies should incorporate neutralization assays, antigen-specific memory B-cell assays, antibody durability, BCR repertoire sequencing, and somatic hypermutation analysis where feasible [21,47,48].

The most informative design would compare patients receiving PD-1/PD-L1 blockade with matched cancer controls receiving chemotherapy, targeted therapy, or observation. Samples should be collected before therapy, during therapy, at treatment discontinuation, and during survivorship. Infection outcomes should be adjudicated carefully, distinguishing bacterial, viral, fungal, mycobacterial, opportunistic, and microbiologically unconfirmed events. Exposure to steroids, infliximab, mycophenolate, rituximab, IVIG, antibiotics, and hospitalization should be treated as time-dependent covariates.

**Figure 4:**
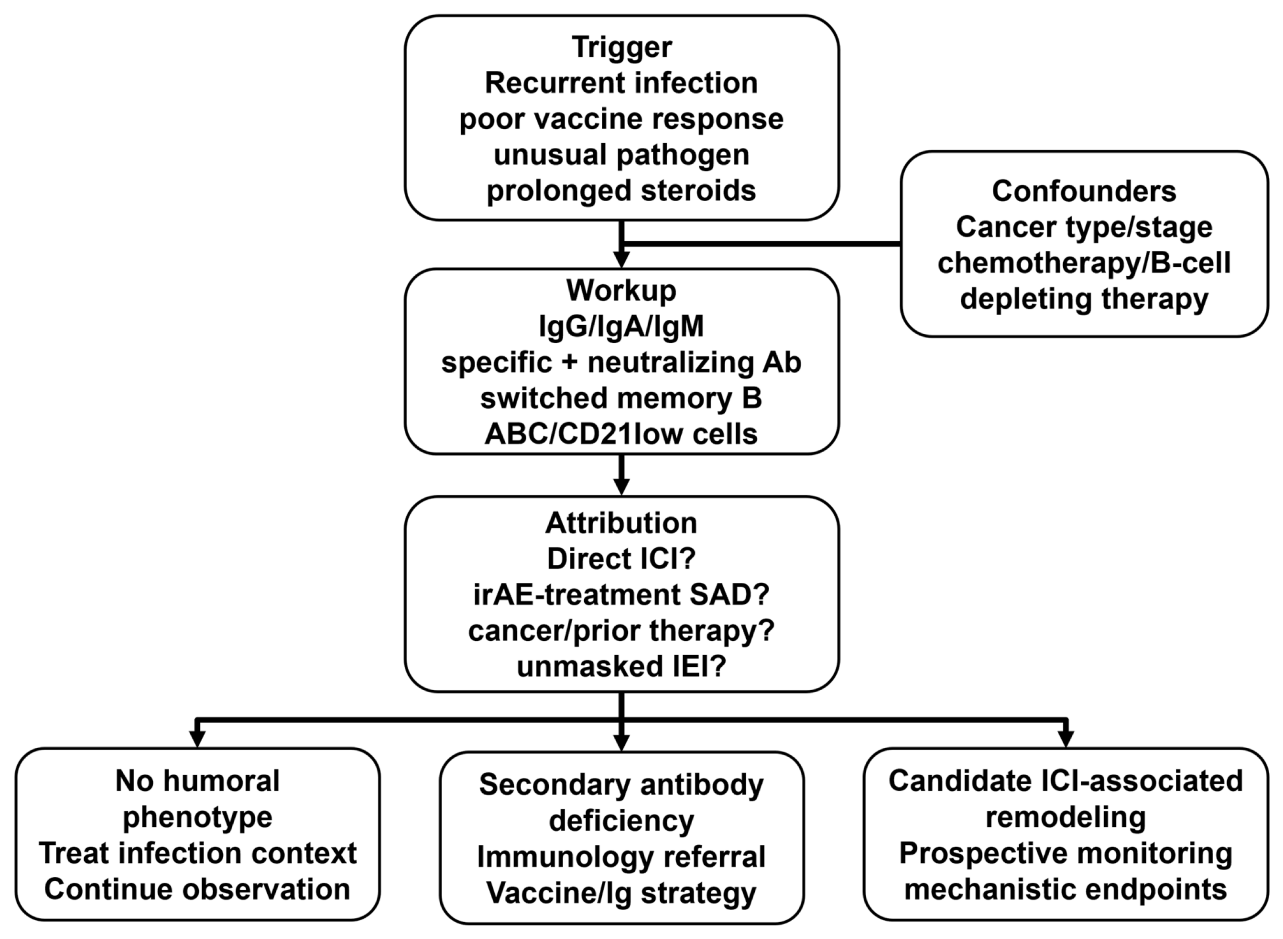
Risk-adapted clinical and research workflow for suspected humoral immune dysfunction after immune checkpoint inhibitor therapy. This proposed workflow outlines a selective approach to evaluating possible humoral immune remodeling or secondary antibody deficiency in patients exposed to long-term immune checkpoint inhibitor therapy. Evaluation is triggered by clinical features such as recurrent infections, poor vaccine response, unusual pathogens, or prolonged corticosteroid exposure. Before attributing the phenotype to checkpoint blockade, major confounders should be reviewed, including cancer type and stage, prior chemotherapy, B-cell-depleting therapy, baseline immune history, protein loss, and immune-related adverse event treatment. The suggested workup includes quantitative IgG, IgA, and IgM; pathogen- or vaccine-specific binding antibodies; neutralizing antibody assays where available; class-switched memory B cells; ABC/CD21low B cells; and infection adjudication. Attribution should then distinguish direct ICI-associated humoral remodeling from irAE-treatment-associated secondary antibody deficiency, cancer- or prior-therapy-related immune deficiency, or an unmasked inborn error of immunity. Possible outputs include continued observation when no humoral phenotype is found, immunology referral and vaccine or immunoglobulin strategy when secondary antibody deficiency is present, or prospective monitoring with mechanistic endpoints when candidate ICI-associated remodeling is suspected. ABC, age-associated B cell; Ab, antibody; CD21low, B cells with low CD21 expression; ICI, immune checkpoint inhibitor; IEI, inborn error of immunity; Ig, immunoglobulin; IgA, immunoglobulin A; IgG, immunoglobulin G; IgM, immunoglobulin M; irAE, immune-related adverse event; SAD, secondary antibody deficiency.

A useful endpoint structure should synthesize clinical and immunological domains: recurrent or severe infection burden; quantitative immunoglobulin decline; impaired vaccine response or waning; reduced class-switched memory B cells; ABC expansion or CD21low skewing; and exposure-adjusted attribution to direct ICI effect, irAE-treatment-related secondary antibody deficiency, malignancy-related immune deficiency, or unmasked primary immune dysregulation. This composite framework addresses the limitation that IgG concentration alone may obscure qualitative antibody failure (Figure 4).

Research gaps should be prioritized according to clinical impact and feasibility rather than listed only as general uncertainties. Table 6 summarizes the most actionable gaps for the next generation of clinical and translational studies.

Pharmacovigilance and real-world database studies can help generate hypotheses, but they should not substitute immunophenotyped cohorts. Electronic health record analyses could identify recurrent infection phenotypes and immunoglobulin testing patterns, while prospective studies can establish causality and mechanism. Mechanistic studies should also test whether anti-PD-1 and anti-PD-L1 antibodies differ in their effects on B-cell activation, Tfh help, PD-L2 compensation, and antibody durability. The inherited PD-L1 deficiency data suggest that ligand and receptor disruption are not immunologically equivalent [15], an issue that has received little attention in clinical immunomonitoring.

## 11. Conclusions

Several points are now reasonably well established. PD-1/PD-L1 signaling is not only an inhibitory pathway in exhausted antitumor T cells. Human inherited PD-1 and PD-L1 deficiencies show that this axis also contributes to immune homeostasis [15,19], and recent mechanistic data implicate it in memory B-cell development and antibody responses [16]. Vaccine studies further show that ICI therapy can alter Tfh and B-cell dynamics without necessarily abolishing serologic responses [17,18,21,34,35].

The author’s interpretation is that humoral immune remodeling is a plausible but not yet proven consequence of long-term PD-1/PD-L1 blockade. The strongest evidence supports biology and mechanism; the clinical evidence remains incomplete. Current data do not define a common ICI-induced antibody-deficiency syndrome and do not justify universal immunoglobulin or B-cell-subset screening. They do justify careful evaluation of selected patients with recurrent infections, unusual pathogens, poor vaccine responses, prolonged immunosuppression, hematologic malignancy, prior B-cell-directed therapy, or other features suggesting secondary antibody deficiency.

The next step for the field is prospective, controlled, immunophenotyped survivorship research. Future studies should measure antibody quantity, antibody function, B-cell memory, ABC/CD21low skewing, infection burden, and treatment confounders from baseline through survivorship. Such studies would allow immune activation, immune dysregulation, and immune deficiency to be distinguished rather than collapsed into a single category. Until then, “humoral remodeling” should be used as a testable framework: useful for organizing evidence and directing monitoring in high-risk patients, but not as a settled clinical diagnosis.

## Figures and Tables

**Table 1:** Summary of the major differences between inherited pathway deficiency and pharmacologic blockade.

Dimension	Inherited PD-1/PD-L1 Deficiency	Therapeutic PD-1/PD-L1 Blockade	Interpretive Implication
Timing	Lifelong, often pediatric; affects immune development	Usually adult-onset; applied to an already formed immune system	Genetic disease can include developmental immune effects not expected after adult therapy
Completeness	Complete or near-complete receptor/ligand loss	Partial, intermittent, dose-dependent receptor or ligand blockade	Clinical ICI effects may be subtler and exposure-dependent
Host context	Rare inborn error of immunity, often with severe immune dysregulation	Cancer patients with age, tumor burden, prior therapies and comorbidities	Confounding is central in clinical interpretation
Pathway target	PDCD1 or CD274 loss, depending on genotype	Anti-PD-1 or anti-PD-L1 antibody exposure	Receptor and ligand blockade may not be immunologically equivalent
Compensation	Depends on the molecular lesion; PD-1 loss blocks PD-L1 and PD-L2 signaling	PD-L2 signaling may remain available during PD-L1 blockade	Anti-PD-1 may have broader pathway effects than anti-PD-L1
Inference	Strong biological plausibility	Clinical hypothesis requiring prospective testing	Genetic evidence supports mechanism, not incidence or causality in ICI-treated patients

**Table 2:** Human PD-1 and PD-L1 deficiency and B-cell remodeling as biological anchors for ICI-associated humoral immune dysregulation.

Condition	Gene/Pathway	Core Clinical Phenotype	Humoral/B-Cell Relevance	Implication for ICI Review
Inherited PD-1 deficiency	PDCD1/PD-1 receptor loss	Early autoimmunity, tuberculosis, fatal pulmonary autoimmunity reported [19]	Reduced memory B cells and impaired antibody responses reported in later mechanistic work [16]	Complete PD-1 pathway disruption produces immune dysregulation rather than simple immune activation
Inherited PD-L1 deficiency	CD274/PD-L1 ligand loss	Neonatal-onset type 1 diabetes; clinically less severe than PD-1 deficiency in one reported family [15]	Reduced memory B-cell and antibody-response defects reported in combined PD-1/PD-L1-deficiency analyses [16]	Receptor versus ligand blockade may differ; PD-L2 compensation warrants further study
PD-1/PD-L1 deficient mice and cellular systems	Pdcd1, Cd274, Pdcd1lg2 models and ex vivo blockade	Experimental immune dysregulation and altered Tfh/B-cell biology	B-cell-intrinsic and extrinsic mechanisms; c-Myc-linked antibody production defects [16]	Mechanistic hypotheses should be tested prospectively in ICI-treated cohorts
Age-associated B-cell expansion during ICI therapy	ABC/CD11c + T-bet + B-cell remodeling	Observed in ICI-treated cancer patients in COVID-19 vaccine studies	Higher baseline ABC frequency correlated with reduced antigen-specific memory B cells and neutralizing capacity [21]	Supports qualitative humoral failure as a distinct phenotype beyond low total IgG

**Table 3:** Proposed diagnostic protocol for suspected ICI-associated secondary antibody deficiency or humoral remodeling.

Domain	Minimum Data to Collect	Purpose
Baseline immune status	Pre-ICI IgG, IgA, IgM; previous infection history; vaccine history	Distinguish pre-existing deficiency from treatment-emergent changes
Humoral quantity	IgG, IgA, IgM, IgG subclasses; serum protein and albumin	Identify hypogammaglobulinemia and protein-loss confounders
Humoral function	Pneumococcal, tetanus, diphtheria, influenza and SARS-CoV-2 binding antibodies; neutralization and durability when available	Assess protective antibody quality and vaccine responsiveness beyond seroconversion
B-cell phenotype	Naive B cells, unswitched memory, class-switched memory, plasmablasts, CD21low cells, ABCs/CD11c + T-bet + cells, antigen-specific memory B cells if feasible	Localize the defect within memory formation, differentiation, atypical remodeling, or ABC expansion
Treatment confounders	Chemotherapy, radiation, anti-CD20 therapy, corticosteroids, infliximab, mycophenolate, tacrolimus, rituximab	Separate direct checkpoint effects from therapy-induced secondary immunodeficiency
Infection adjudication	Pathogen, site, recurrence, microbiologic confirmation, hospitalization, antibiotic exposure	Convert nonspecific infection labels into interpretable immune phenotypes
Standards mapping	ESID/CVID-like domains, secondary hypogammaglobulinemia criteria, exclusion of secondary causes, immunology referral when indicated	Avoid misclassifying acquired or treatment-related hypogammaglobulinemia as primary CVID

**Table 4:** Mapping of proposed ICI-associated humoral dysfunction categories to established immunological frameworks.

Proposed ICI Category	Closest Existing Framework	Suggested Minimum Evidence Required
Direct checkpoint-blockade-associated humoral dysregulation	Acquired CVID-like or unclassified antibody-deficiency phenotype, not primary CVID unless pre-existing criteria are met	Temporal association with PD-1/PD-L1 blockade; poor vaccine response or impaired neutralization; reduced switched memory B cells and/or ABC expansion; exclusion of therapy, malignancy, protein loss, and primary causes
irAE-treatment-associated secondary antibody deficiency	Secondary hypogammaglobulinemia/secondary antibody deficiency	Prolonged corticosteroids, rituximab, mycophenolate, infliximab, cyclophosphamide, or other immunosuppressants; low Ig or impaired function emerging after immunosuppression
Cancer-associated or prior-treatment-related immune deficiency	Secondary antibody deficiency due to malignancy, chemotherapy, anti-CD20 therapy, transplant, protein loss, or marrow failure	Relevant cancer or therapy exposure; baseline or pre-ICI immune compromise; exclusion of direct ICI attribution
Unmasked inborn error or primary immune dysregulation	ESID/IUIS inborn error framework	Pre-existing recurrent infections, autoimmunity, family history, low baseline immunoglobulins, abnormal B-cell phenotype, and genetic testing when clinically indicated

**Table 5:** Evidence strength for ICI-associated humoral immune remodeling.

Evidence Domain	Current Strength	Interpretation	Main Limitation
Inherited PD-1/PD-L1 deficiency	Strong biological evidence	Shows the pathway is required for immune homeostasis and antibody/memory B-cell biology [15,16,19]	Not equivalent to adult therapeutic blockade
Mechanistic human/mouse studies	Strong mechanistic plausibility	Supports B-cell-intrinsic, Tfh-linked, and c-Myc-linked mechanisms [16,20]	Translation to cancer patients requires direct testing
Vaccine studies	Moderate and mixed clinical evidence	Seroconversion is often preserved; Tfh/B-cell dynamics and neutralization may differ [17,18,21,34,35]	Heterogeneous endpoints and short follow-up
Vaccination-survival analyses	Hypothesis-generating clinical evidence	Vaccination may potentiate antitumor immunity during ICI therapy [36]	Retrospective design and potential confounding
Infection/pharmacovigilance studies	Signal-generating evidence	Identify patterns and unusual pathogens [37,38]	Cannot estimate incidence or establish mechanism
Case-level reports	Lowest causal certainty	Illustrate adjacent or confounded immune-deficiency phenotypes [39,40,41,42,43]	Sparse, heterogeneous, and confounded

**Table 6:** Priority research gaps for humoral immune remodeling during ICI therapy.

Priority	Research Question	Why it Matters	Feasibility
High	Does long-term anti-PD-1/PD-L1 therapy reduce class-switched memory B cells?	Directly tests the central hypothesis	High
High	Are neutralizing antibodies impaired despite seroconversion?	Clinically actionable for vaccine protection	High
High	How much of the phenotype is driven by steroids or irAE immunosuppression?	Major confounder and modifiable risk	High
High	Do anti-PD-1 and anti-PD-L1 agents differ in humoral effects?	Receptor versus ligand biology may differ; PD-L2 compensation is relevant	Moderate
Moderate	Are mucosal IgA responses altered?	Relevant to respiratory and gastrointestinal infections	Moderate
Moderate	Are changes reversible after ICI discontinuation?	Critical for survivorship counseling	Moderate
Moderate	Do ABC/CD21low cells predict infection or vaccine failure?	Potential biomarker for risk-adapted monitoring	High
Exploratory	Does BCR repertoire quality or somatic hypermutation change?	Provides mechanistic depth	Lower

## Data Availability

Not applicable; this article is a narrative review of the previously published literature. During manuscript preparation and revision, the author used AI-assisted tools (ChatGPT 5.5 and JenniAI Plus) for language polishing, structural organization, figure development, and formatting support. The author critically reviewed, revised, verified, and approved all content, references, interpretations, and conclusions.

## Abbreviations

ABC	age-associated B cell
AID	activation-induced cytidine deaminase
APC	antigen-presenting cell
BCR	B-cell receptor
CSR	class-switch recombination
CVID	common variable immunodeficiency
ESID	European Society for Immunodeficiencies
ICI	immune checkpoint inhibitor
IEI	inborn error of immunity
Ig	immunoglobulin
irAE	immune-related adverse event
PD-1	programmed cell death protein 1
PD-L1	programmed death-ligand 1
SAD	secondary antibody deficiency
SHM	somatic hypermutation
Tfh	T follicular helper cell
